# Structural and Functional Dynamics of Dehydrins: A Plant Protector Protein under Abiotic Stress

**DOI:** 10.3390/ijms19113420

**Published:** 2018-10-31

**Authors:** Zhengyang Yu, Xin Wang, Linsheng Zhang

**Affiliations:** College of Life Science/State Key Laboratory of Crop Stress Biology for Arid Areas, Northwest A&F University, Yangling 712100, China; gomopfish@163.com (Z.Y.); wxindw@163.com (X.W.)

**Keywords:** dehydrin, abiotic stress, protein structure, gene expression, intrinsically disordered proteins

## Abstract

Abiotic stress affects the growth and development of crops tremendously, worldwide. To avoid adverse environmental effects, plants have evolved various efficient mechanisms to respond and adapt to harsh environmental factors. Stress conditions are associated with coordinated changes in gene expressions at a transcriptional level. Dehydrins have been extensively studied as protectors in plant cells, owing to their vital roles in sustaining the integrity of membranes and lactate dehydrogenase (LDH). Dehydrins are highly hydrophilic and thermostable intrinsically disordered proteins (IDPs), with at least one Lys-rich K-segment. Many dehydrins are induced by multiple stress factors, such as drought, salt, extreme temperatures, etc. This article reviews the role of dehydrins under abiotic stress, regulatory networks of dehydrin genes, and the physiological functions of dehydrins. Advances in our understanding of dehydrin structures, gene regulation and their close relationships with abiotic stresses demonstrates their remarkable ability to enhance stress tolerance in plants.

## 1. Introduction

Environmental stress such as drought, high salinity, and extreme temperatures can negatively affect plant growth and development. To confront various environmental constraints, plants have evolved systematic defense mechanisms to decrease the loss of intracellular water in order to maintain the integrity of cell structures.

Late embryogenesis abundant (LEA) proteins, initially discovered in cotton [1], have been found to accumulate in many plant species during environmental stress and seed development stage. Subsequent studies of LEA proteins indicate their involvement in improving stress resistance in plants. LEA proteins are highly hydrophilic, and their structural analysis implies that they are intrinsically disordered proteins (IDPs), which provide a flexible property to interact with metal ions and biomolecules [2,3]. There are 7 groups of LEA proteins based on sequence similarity and structural property [4]. Although variations have been observed between different LEA groups, they share qualities of high hydrophilicity and Gly richness [5,6,7].

The most commonly described LEA proteins are known as dehydrins (or group II LEA). Like typical LEA proteins, dehydrins are highly hydrophilic and thermostable. Dehydrins mainly accumulate in the later stages of embryogenesis in response to various environmental stresses [4]. According to recent studies, dehydrins have been found widely distributed in angiosperm [5,6,7,8,9,10], gymnosperm [11,12,13] and bryophyte [14,15] plants.

The expression of dehydrins can be induced by various abiotic factors and phyto-hormones [6,7,16,17,18]. Since some dehydrins can respond to abscisic acid (ABA), they are referred to as Response to ABA (RAB) proteins. For instance, the expression of a wheat dehydrin WZY2 can be stimulated by drought, salinity and ABA [9,17]. The main functions of dehydrins have been discovered to participate significantly in stabilizing membranes, enzymes, and nucleotides in cells under abiotic stresses. Studies on transgenic plants have also proved that overexpression of dehydrins could enhance the tolerance to various abiotic stresses. Two dehydrins from the moss *Physcomitrella patens*, PpDHNA and PpDHNC, have been shown to improve the stress resistance to salinity and drought in transgenic Arabidopsis. The transgenic lines also exhibited strong scavenging ability for reactive oxygen species (ROS) with enhanced content of superoxide dismutase (SOD) and catalase (CAT) [14]. On the other hand, knockout of PpDHNA in *Physcomitrella patens* mutant lines severely impaired their ability to recover from salt and osmotic-stress treatments [19]. Similar to PpDHNA, overexpression and RNAi assays in transgenic *Arabidopsis* imply that cold-regulated (COR) proteins, dehydrins (COR15A and COR15B) are necessary for full cold acclimatization as well [20].

**Figure 1:**
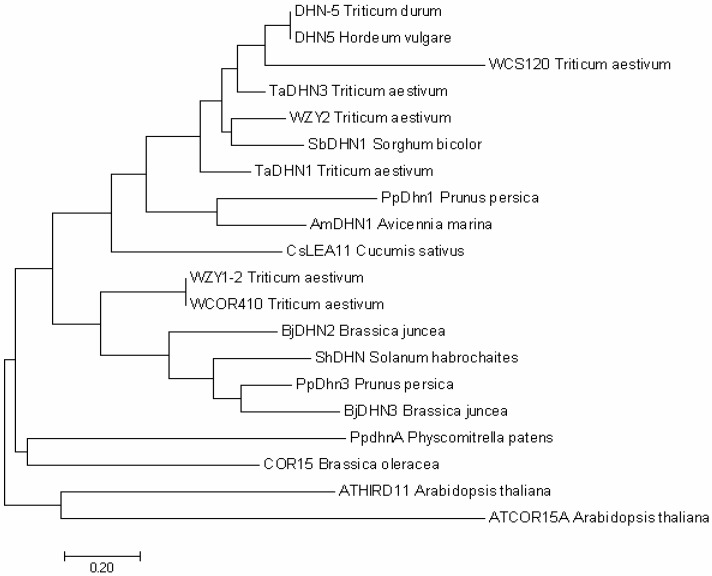
Phylogenetic analysis of well-studied dehydrins. Phylogenetic relationship of well-studied dehydrins. The bootstrapped tree with 1000 replicates was constructed using MEGA 7 tools.

Several studies on differential expression of dehydrins in frost-tolerant and frost-susceptible wheat genotypes have revealed that transcripts of many dehydrins are higher in frost-tolerant wheat, such as WCS120, Wdhn13 [21,22]. Similar to WCS120, a dehydrin from barley (DHN5) was also accumulated in higher amounts in winter cultivars than spring ones [23,24]. These results suggest a significant correlation between dehydrins and acquired frost tolerance (FT) of plants, hence these dehydrins can also be considered promising FT markers. We also draw a phylogenetic analysis tree of well-studied dehydrins in this article (Figure 1).

Although the detailed and overall mechanisms in dehydrin functioning are still ambiguous, some protective models have been proposed to elucidate their role in different aspects, and will be discussed in this review. In addition, advances in dehydrin structures, gene regulations and their close relationships with abiotic stresses will be elucidated in accordance with recent studies.

## 2. Molecular Structure of Dehydrins

Most dehydrins are low molecular weight proteins, and their molecular weights range from 9 kD to 200 kD [25]. Dehydrin protein families contain three conserved motifs, named K-, Y- and S-segments. Among the conserved motifs, a Lys-rich residue called K-segment is prevalent in all dehydrins. In angiosperms, the common sequence of K-segment is EKKGIMDKIKEKLPG, while in gymnosperms, dehydrins share a more variable sequence: (Q/E)K(P/A)G(M/L)LDKIK(A/Q)(K/M)(I/L)PG [26,27]. K-segment is usually located near the C-terminal and participates in forming a class of A2 amphipathic α-helix [28]. Some dehydrins possess more than one K-segment, and they share distinct amino acid sequences. Although the amino acid position in K-segment varies slightly, conserved residues still exist. The conserved residues are Lys-Ile-Lys-Glu in the core, Glu-Lys-Lys in the N-terminal and Lys-Leu-Pro-Gly in the C-terminal of this segment [25]. Y-segment (DEYGNP) is an N-terminal conserved sequence, and it is homologous to the nucleotide-binding of chaperones of bacteria and plants [29]. S-segment is a serial of the serine cascade sequence (SSSSSSSD), and this segment often has one copy in the dehydrins. Based on their conserved segments, dehydrins are usually classified into five subfamilies: KnS, YnSKn, Kn, YnKn, and SKn [30].

In addition, some untypical segments also appear in many dehydrins, and these segments exhibit various properties and functions. One segment that has been found is a polyhistidine- or histidine-rich motif, which contributes to binding ability of dehydrin with ligands. OpsDHN1 from *Opuntia streptacantha* with a polyhistidine motif exhibited self-dimerizing via a split-ubiquitin yeast two-hybrid system; subsequent deletion of this motif results in loss of self-interaction [31]. Three dehydrins from *Arabidopsis*, AtCOR47, AtERD10, and AtRAB18, also showed formation of homodimeric complexes, as well as heterodimeric complexes with each other [32].

In addition to dehydrin-dehydrin interaction, a histidine-rich motif also participates in dehydrin metal binding and lipid binding. A KS-type dehydrin, AtHIRD11, inhibited generation of reactive oxygen species (ROS) in a Cu-ascorbate system by binding with free Cu^2+^. Meanwhile, dehydrins with His-flanking K-segments are capable of binding to membrane structures [33]. In many YnSKn-type dehydrins, the K-segment and S-segment are linked by a fixed motif, GXGGRRKK (X can be any amino acid), which suggests a potential functional linkage between K- and S-segments. The GXGG motif is highly flexible and participates in the interaction of negatively charged phosphoserines with K-segment, while RRKK motif is considered to be a nuclear localization signal [34,35,36].

Based on circular dichroism (CD), nuclear magnetic resonance (NMR) and Fourier Transform Infrared Spectroscopy (FTIR) analysis, dehydrins are characterized as intrinsically disordered proteins (IDPs) [37,38]. These do not adopt a fixed structure, but contain flexible mobile polypeptides [39,40]. The NMR spectrum provides a general view of IDP structures with respect to the chemical shift values in their secondary structures [41]. The mutual authentication from CD, FTIR, and NMR studies has helped to clearly uncover the transformation of dehydrin secondary structures.

Interaction of dehydrins and their ligands (metals, biomembranes, and proteins/enzymes) could strongly induce the formation of the ordered protein structure of dehydrins. According to Hara et al., (2013), a KS-type dehydrin AtHIRD11 showed an obvious signal minimum near 200 nm under CD determination, which represents a random coil. However, disordered conformation was attenuated by Cu^2+^ [42]. A similar conformational change was also revealed in TsDHN-1 and TsDHN-2 with Zn^2+^ via CD and FTIR assay. In addition, this phenomenon is also associated with variation in temperature [43]. Lactate dehydrogenase (LDH) is widely used in dehydrin cryoprotective assays [7,9,16,44,45]. It has been reported that K2 and YSK2 dehydrin were able to protect LDH activity better than BSA. Compared to K2, YSK2-type dehydrin provided a 50% higher LDH activity at a 2-fold lower protein concentration, suggesting that larger proteins could provide more efficient protection [45].

In another study, a small model dehydrin *Vitis Riparia* K2 was selected to examine protection of membranes under freeze-thaw stress with CD and NMR spectrum. The results showed the ability of this protein to bind with liposomes which contain phosphatidic acid, leading to protection of liposomes under freeze-thaw stress. Further analysis implies that K2 dehydrin did not significantly affect liposome surface accessibility or lipid mobility; however, presence of K2 could decrease membrane transition temperature by up to 3 °C. Experiments involving NMR showed that conserved lysine-rich segments participated in the binding of dehydrin to membranes and poorly conserved φ-segments, and did not show any relevance in dehydrin binding and protection [46].

The results are consistent with previous studies that revealed structure of dehydrins could become ordered or partially ordered through binding with ligands [15,44,47]. The lack of defined secondary structures may contribute to flexible binding properties of dehydrin with other ligands other than Cu^2+^ and Zn^2+^, such as lipid membrane and dehydrins [31,33].

## 3. The Regulation of Dehydrin Genes under Abiotic Stresses

**Figure 2:**
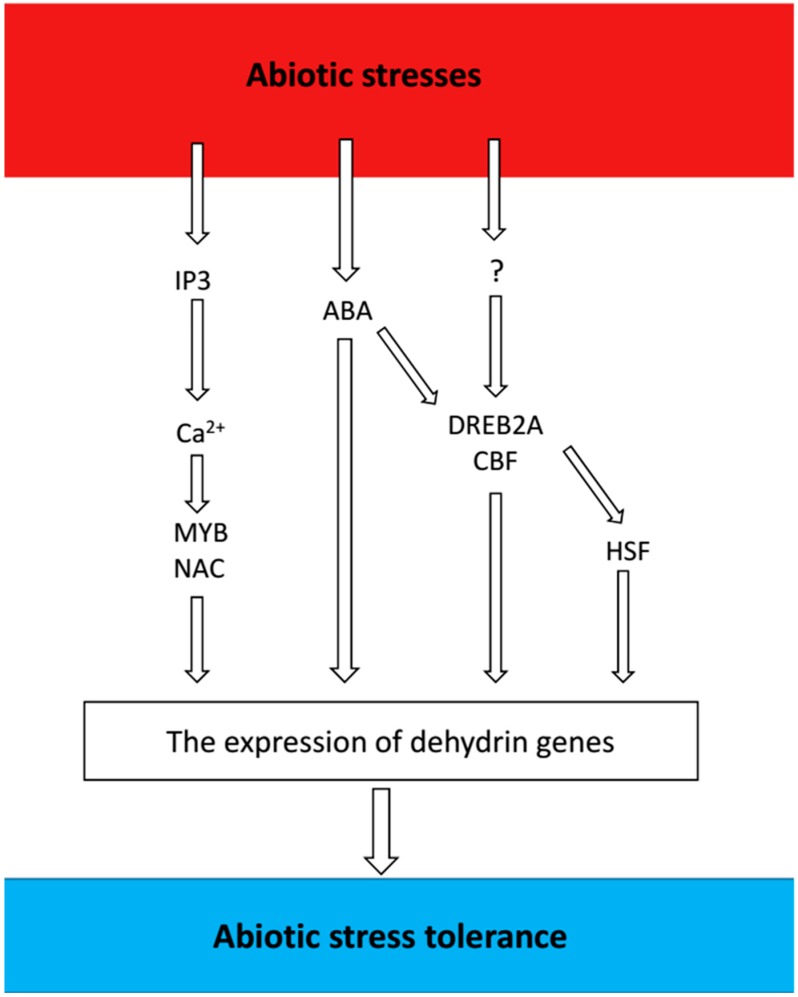
Overview of signaling pathways and factors involved in signaling regulation of dehydrin genes. The figure shows the signaling components involved in the abiotic stress responses that are described in the text. Question marks represent as-yet-unidentified factors in the corresponding signal transduction pathways.

The expression of dehydrins is strongly induced by adverse environmental factors. Some dehydrins share intricate response mechanisms, and their expressions can be triggered by multiple stresses. Abiotic stresses can trigger rapid accumulation of dehydrins in plants through multiple signaling pathways (Figure 2).

### 3.1. Drought Stress and Dehydrins

Drought is one of the most prevalent constraints in plant development due to severe restriction of cell extension, reduction of cell water content, and effects on photosynthetic activity. In addition, drought stress can induce secondary stresses, such as osmotic and oxidative stress [48]. *ShDHN* is a gene whose expression reaches its maximum level (almost 12-fold-changes) in 6 h under drought conditions. Similarly, *SiDhn2* expression increases 12-fold after 3 h of dehydration stress [49,50]. However, dehydrin *WZY2* gene shows lower response to dehydration for its maximum expression level at 24 h after stress [17]. These findings suggest that different dehydrins may respond at different time intervals in order to mediate distinct stages of drought stress.

Abscisic Acid (ABA) acts as a vital mediator in plant abiotic stress, regulates expression of drought stress-related genes, and protects cells against damage due to abiotic stress leading to increased stress tolerance. Presence of ABA-responsive *cis*-elements (ABRE) in promoters of dehydrin genes enable them to participate in the ABA-dependent pathway. So far, several ABRE motifs are found in dehydrin gene promoters in wheat (CCACGTGTC/GACACGTGG) [51], maize (CCGACGCG/CGCGTCGG) [52], rice (CCGACGCG/CGCGTCGG) [53] and barley (CCAACGCG/CGCGTTGG) [54]. Many dehydrin genes are induced by exogenous ABA treatment, as reported in various studies [17,49]. In ABA-dependent signaling, ABRE-binding protein/ABRE-binding factors (AREB/ABFs) play a pivotal role in regulating the expression of ABA-dependent genes. Moreover, the AREB/ABF family belongs to group A of the basic leucine zipper (bZIP) transcription factor (TF) family, and studies have revealed that overexpression of AREB in the transgenic plant could significantly activate the expression of dehydrins [55].

Some dehydrins possess dehydration-responsive elements (DRE, A/GCCGAC motifs) with or without ABRE motifs. The DRE motif is one of the core domains for dehydrin genes in responding to drought stress in the ABA-independent pathway. Promoters of peach dehydrins PpDhn1 and PpDhn3 share a typical CRT element (known as C-repeats, RCCGAC) upstream of the TATA box [56]. Additionally, some SKn- and YnSKn-type dehydrins in plants also possess de novo DRE/CRT motifs [57]. Previous studies have provided evidence that transcription factors DREB1 and DREB2 are capable of binding to the DRE element of *Arabidopsis* rd29A in order to mitigate drought stress [58,59]. Gupta et al. found that in transgenic tobacco, a *Salicornia brachiate* DREB factor could significantly up-regulate the expressions of NtERD10B and NtERD10D [60]. Furthermore, overexpression of *Vigna radiata* DREB gene *VrDREB2A* in transgenic *Arabidopsis* also enhances the expression of *AtCOR15A*, *AtCOR15B*, *AtKIN1*, *AtRD17*, *AtRD29A*, and *AtRD29B*, indicating an important role of the DREB family in the dehydrin regulation pathway under drought stress [61]. Kim et al. discovered binding activities of three AREB members (AREB1, AREB2, and ABF) to ABRE *cis*-elements in DREB2A promoter, which is also necessary for DREB2A expression under osmotic stress. Furthermore, in plant vegetative tissues, AREB was also shown to interact with DREB in ABA-related gene expression under dehydration conditions [62,63]. VvDHN1 from *V. vinifera* leaves is hypersensitive to both drought and ABA; after four days of drought stress and eight hours of ABA treatment, the expression levels of the *VvDHN1* gene reach about a 350-fold change and a 170-fold change, respectively [18]. These results imply that dehydrins are participate broadly in complicated crosstalk signaling networks under the regulation of drought-related TFs.

### 3.2. Cold Stress and Dehydrins

Cold stress can directly influence the fluidity of plant cell membrane and enzyme activity, resulting in metabolic disturbance, photosynthesis inhibition, material transportation disorder and, finally, damage to plants [64]. Cold stress can induce *ShDHN* and *WZY2* dehydrin, with more than 40-fold and 80-fold changes in gene expression levels, respectively. Moreover, promoters of these dehydrin genes contain low temperature-responsive elements (LTREs), which play a crucial role in their response to cold stress. For example, ShDHN promoter contains two LTRE elements [49]. However, the regulation of LTREs still needs to be clarified. In addition to drought stress response, DREs also participate in cold stress signaling. In *Arabidopsis*, overexpression of AtDREB1A or AtDREB2A induces cold stress-related genes such as rd29A and COR47 under cold stress [63].

C-repeat response element binding factors (CBFs), belonging to the DREB family, contain AP2 DNA binding domains. AtCBF1, AtCBF2, and AtCBF3 can be up-regulated more rapidly and significantly by cold stress than ABA and dehydration [65,66]. Previous studies have shown that some CBFs are specifically induced by cold stress. Dehydrin gene promoters from numerous species contain the low-temperature response element CRT. Study on CBFs has revealed its functions in binding to DRE or CRT motifs [67]. In addition, several DREB promoters have been characterized that contain ABRE motifs, including MACGYGB and CGTGG, in *Sorghum*, and over-representation of motifs related to other phytohormone signalings in DREB promoters has also been reported [68]. Based on microarray analyses, several dehydrins induced by cold and dehydration have been characterized in *Arabidopsis*, rice, and soybean [69].

Interestingly, some evidence showed that dehydrin gene promoters containing DREs did not respond to drought and/or cold stress. PpdhnA promoter contains one DRE and several ABRE motifs, but qRT-PCR results showed that this gene could not be induced by cold stress [15]. This reveals a complicated mechanism of cold/drought regulation in dehydrin genes which needs further elucidation to understand the related networks involved.

### 3.3. Heat Stress and Dehydrins

Heat stress is an intricate process that often directly causes protein denaturation and aggregation, and increases membrane lipids fluidity [70]. Evidence from many studies has revealed a strong association between dehydrins and plant tolerance to heat stress. For instance, dehydrin CsLEA11 and WZY2 proteins were able to protect both recombinant *E. coli* and activity of the LDH enzyme under heat stress [9,16]. Dehydrin DHN-5 from wheat was found to play a relevant role in protecting enzyme β-glucosidase (bglG) against heat stress. Truncation assay of DHN-5 indicated that K-segments were vital to the thermal protection of dehydrins [71,72].

In wheat, promoters of dehydrin TaDHN1 and TaDHN3 genes possess heat stress elements (HSEs), that are involved in heat stress response [73]. HSEs can be specifically recognized by heat stress transcription factors (HSFs) due to its conserved N-terminal DNA binding domain (DBD). However, there is little evidence showing that HSEs is also present in other known promoters of dehydrins.

Other than HSEs, promoters of dehydrins often possess DREs with intricate regulation mechanisms under heat stress. In Arabidopsis, the DRE-binding protein 2A (DREB2A) could directly regulate expression of *AtHSFA3* through binding two DRE elements in the AtHSFA3 promoter [74]. As mentioned above, the DREB protein family also participates in the regulation of dehydrins under drought and cold stresses, implying that they may share similar strategies with plant tolerance to drought, cold and heat stresses.

### 3.4. Salt Stress and Dehydrins

The plant tolerance mechanism to salt stress has been widely studied and extensively characterized in many plants in which both ABA-dependent and ABA-independent signaling pathways are involved [75,76,77]. High salinity concentration causes cell osmotic change and water loss, and eventually damages the cell. Salt tolerance mechanisms involve maintaining the homeostasis of cellular ions and osmotic adjustment and ROS scavenging [78]. Overexpression of a salt stress-induced dehydrin DHN-5 from Durum wheat was found to enhance salt tolerance in transgenic *Arabidopsis*. Furthermore, numerous dehydrin genes have been identified to respond to salt stress, revealing the relationship between salt stress and dehydrin on a larger scale, as reported by omics studies [79,80,81]. Further overexpression and interference with dehydrin genes in transgenic plants validated association of dehydrin with salt tolerance in plants [10,19,49,81,82,83,84].

The osmotic pressure induced by salt stress leads to an increase in the concentration of Ca^2+^ and inositol 1,4,5-triphosphate (IP3) in the cytosol [85]. As secondary messengers, Ca^2+^ and IP3 then activate the mitogen-activated protein kinase (MAPK) cascades to regulate phosphorylation of various transcription factors including CBF/DREB, ABF, bZIP, Myc/MYB, and NAC (NAM, ATAF, CUC) factors [86]. Analyses of known dehydrin promoters have also shown many vital *cis*-elements [17,57]. One putative DRE element (ACCGAC) and one putative MYB transcription factor binding element (YAACKG/CANNTG), corresponding to MYB-binding sequences in *Arabidopsis*, were predicted in an isolated dehydrin *AmDHN1* promoter [87]. However, there is no clear evidence regarding the interaction between these transcription factors and dehydrin promoters.

These results reflect the intricacy of the expression pattern of dehydrins under drought, cold and salt stresses. These motifs greatly contribute to the regulation of dehydrin genes under various abiotic stresses. Additionally, some other phytohormone responsive elements are also found in dehydrin promoters. However, explicit regulation of dehydrin genes requires further studies to elucidate precise mechanisms involved.

### 3.5. Heavy Metal Toxicity and Dehydrins

Studies on the functional roles of dehydrin in heavy metal toxicity are limited in plants. However, there is evidence that implies important role of dehydrins in plant against heavy metal stress. An isolated SK2-type dehydrin gene PvSR3 was highly induced by heavy metal stress [88]. Similarly, two *Brassica juncea* dehydrin genes, *BjDHN2* and *BjDHN3*, were both induced under heavy metal stress. Moreover, overexpression of *BjDHN2* and *BjDHN3* in transgenic tobacco plants significantly enhanced their tolerance to heavy metal [89]. Dehydrins exhibited metal binding behavior in in vitro experiments, which uncovers potential mechanisms of plant tolerance to heavy metal. A binding property test of a dehydrin CuCOR15 implemented using immobilized metal ion affinity chromatography (IMAC) demonstrated that CuCOR15 was capable of binding to Fe^3+^, Co^2+^, Ni^2+^, Cu^2+^ and Zn^2+^ ions. Further truncated assay also indicated that HKGEHHSGDHH was the core sequence for binding [90]. Another in vitro study on dehydrin AtHIRD11, showed a similar metal binding behavior with CuCOR15. Subsequent amino acid mutation assay suggested the presence of histidine in AtHIRD11, which has an important role due to its metal binding property. Since both CuCOR15 and AtHIRD11 are radical-scavenging proteins, the results imply that dehydrins bind metals through a specific sequence containing histidine to reduce metal toxicity in plant.

An understanding of regulatory networks is essential for elucidating a full vision of associated links between heavy metal tolerance and dehydrins. DREB transcription factor family can play important roles in several abiotic stresses, including drought, freezing and heavy metal. Overexpression of *DREB* in transgenic plants showed activated expression of dehydrins such as COR15a, RD29A, RD29B, leading to improved tolerance to drought, heat and heavy metals [91,92,93,94,95]. Several pieces of evidence have unveiled close relationships between dehydrins and plant tolerance to heavy metals. However, further studies about interaction of dehydrin promoters and transcription factors are extremely necessary for deep understanding of mechanism of action.

Here we provide an overview of the dehydrins involved in abiotic stress signaling, as shown in Table 1. Although possible regulatory pathways under various abiotic stresses in plants have not been specifically unified, they still provide credible directions for studies on improving the plant tolerance against stresses.

## 4. Localizations and Functions of Dehydrins

### 4.1. The Localization of Dehydrins

The localization and functional versatility of dehydrins has been widely studied with respect to plant tolerance against multiple abiotic stresses. Dehydrins are ubiquitously localized in different parts of cells, such as cytoplasm, nucleus, chloroplasts, vacuole, endoplasmic reticulum, mitochondria, cytoplasm and cell membranes [9,96,97,98,99,100,101,102]. Some dehydrins are also found to be localized in several parts of cells under abiotic stresses. GFP-fused dehydrin WZY2 is observed in both the nucleus and cytoplasm under cold stress [9]. Correspondingly, DHN24 is detected to be localized in cytosol and close to organelle membranes through immuno-electron microscopy [103].

As mentioned above, histidine-rich motifs in dehydrins may control its nuclear localization. OpsDHN1 is a nuclear-localized protein, whereas deletion of its histidine-rich segment results in significantly lower signal detection in the nucleus of OpsDHN1. Further studies on OpsDHN1 revealed another vital segment of dehydrin, S-segment, which is probably relevant for OpsDHN1 nuclear localization. In silico analysis also predicted a putative nuclear localization signal (NLS) containing partial of OpsDHN1 S-segment using cNLS mapper [97,104]. A report on CuCOR15 suggests that the phosphorylation state of serine in dehydrin S-segment is involved in its translocation to the nucleus. In addition, S-segment is activated by phosphorylation of casein kinase 2 (CK2) in *Arabidopsis* [105]. Meanwhile, phosphorylation of RAB17 was significant in binding nuclear localization signal (NLS) peptides in vitro [106]. As they are localized throughout the cells, dehydrins may provide more extensive support in protecting cell membranes and biomolecules under abiotic stresses.

### 4.2. The Multifunction of Dehydrins

The ubiquitous localization of dehydrins suggests their versatile functions in plant tolerance to abiotic stresses. Transgenic studies on dehydrin function imply a wide consensus that dehydrins are positively associated with tolerance of the plants against abiotic stress. It is reported that PpDHNB can enhance the cold tolerance in transgenic tobacco [15]. Overexpression of *DHN-5* confers salt tolerance to transgenic *Arabidopsis* with higher expression of P5CS, proline, CAT, SOD, and POD [10]. Similar to PpDHNB and DHN-5, ShDHN significantly improves the tolerance of transgenic tomatoes under drought and cold stresses, leading to retention of higher relative water content and less ROS accumulation in leaves. The MDA contents in transgenic lines showed a lower level than that of wild-type tomatoes [49]. On the other hand, silencing of CaDHN1 resulted in lower accumulation of chlorophyll and expression of POD and Mn-SOD in *Capsicum annuum* under multiple abiotic stresses [81]. The above results provide a macroscopic view of dehydrin functions in enhancement of tolerance of plants at physiological level.

Protection of biomolecules is the main function of dehydrins during stress, as revealed by in vitro and in vivo studies. Overexpression of dehydrin genes usually improves the RWC in transgenic plants [49]. Amphipathic α-helix assists in bounding of dehydrin and water in cells [45,46,96,107], while on the other hand, dehydrins can stabilize cell structures and organelles to prevent water loss during stress [108,109]. In wheat dehydrin WZY2, K-segment is essential for the protecting activity of lactate dehydrogenase (LDH) and sustaining the survival rate of transformed *E. coli* under temperature stress [9]. A similar pattern was observed in DHN1a from *Vitis vinifera* and DHN5 from *Rhododendron catawbiense*, the lack of K-segment in which leads to a reduction in the effects of enzyme and bacterial protection [110]. The histidine-rich motifs in some dehydrins have been reported to participate in the binding function. Dehydrin CuCOR15 has been reported to bind DNA, and the presence of Zn^2+^ could strongly promote the ultimate binding [111]. Using an electrophoretic mobility shift assay, dehydrin DHN1a with a histidine-rich motif revealed weak interaction with double-strand DNA fragments. However, none of the divalent metals were capable of enhancing interaction [110].

In addition, some dehydrins with metal-binding capacity are regarded as ROS scavengers under abiotic stresses. In a common ROS-generation system, Cu^2+^ acts as an electron donor, promoting the variation from O_2_ to O_2_^−^, H_2_O_2_ and eventual ·OH in the presence of ascorbate. The addition of dehydrin AtHIRD11 could limit free Cu^2+^ to inhibit ROS generation. Meanwhile, CD spectrum implied a conformational change in AtHIRD11 participating in the inhibition of ROS synthesis [112]. The structural studies within AtHIRD11 suggested a strong relation between conformational changes and metal-chelating in ROS scavenging.

K-segment and histidine-rich motifs play vital roles in interaction, not with only enzymes and DNA, but also with phospholipids, which provide a potential structural foundation in membrane binding under various stresses [38]. A recent study has elucidated the pattern of dehydrin binding with lipid membrane. Dehydrin LTI30 contains K-segment with flanking His residues. NMR spectrum implies that LTI30 are membrane vesicles binding protein and require positively charged residues. Further study also suggested that LTI30 could be modulated by phosphorylation at predicted sites within K-segments. These results imply that protonation of His residues and phosphorylation of K-segments have key roles in lipid vesicle binding of dehydrins [33]. Although not all dehydrins contain histidine-rich motifs, above studies imply an important feature of dehydrin function.

Some possible mechanistic models in dehydrin functioning have been proposed. Dehydrins/LEA proteins may bind to liposomes [20,46,113] or sequester metal ions to form dehydrin-metal ion complex in order to interact with other biomolecules [42,43,47,112]. Recent studies have uncovered that several dehydrins form homo- and heterodimeric complexes, or even multimers, to bind and protect biomolecules for purpose of maintaining the cell and organelle structures and adjusting regular cell processes under adverse conditions of stress.

The capability of dehydrins to bind to multiple ligands provides protection to plants against abiotic stresses. Although the function of K-segment has been widely studied, the physiology of the other two conserved motifs, S- and Y-segments, is still ambiguous. Further studies on the functions of dehydrins are required to strengthen the existing evidence, and supporting data is needed to uncover the potential of dehydrins in plant physiology, especially under stress conditions.

## 5. Conclusions

The interest in exploring the association between dehydrins and abiotic stresses has been increasing recently, as it is very important to elucidate how plants respond to environmental stresses.

The molecular structures, functions and localizations of dehydrins have been widely explored, indicating their potential role in stress tolerance in plants. Potential regulatory mechanisms of dehydrins associated with various abiotic stresses are discussed in this article. However, description of stress-induced regulatory networks of dehydrins is scarce. Further studies on dehydrins should mainly focus on upstream mechanisms, especially with regard to identification of transcriptional cascades and regulatory mechanism of dehydrin genes. This will definitely be helpful in the breeding of stress-tolerant plants in the future to increase global crop production.

## Figures and Tables

**Table 1:** An overview of dehydrins involved in abiotic stress signaling.

Gene Name	Expression Characteristics	Promoter Elements	Species	References
*ShDHN*	Drought, Salt, ABA	ABRE, LTRE, CRT	*Solanum habrochaites*	[49]
*WZY2*	Drought, Cold, ABA	ABRE, LTRE	*Triticum aestivum*	[17]
*rd29A*	Drought, Cold, Salt	DRE	*arabidopsis thaliana*	[58,59]
*PpDhn1*	Cold	ABRE, CRT	*Prunus persica*	[56]
*PpDhn3*	Cold	CRT	*Prunus persica*	[56]
*VvDHN1*	Drought, ABA	ABRE, DRE, HSE, LTRE	*Vitis vinifera*	[18]
*PpdhnA*	(not cold inducible)	ABRE, DRE	*Physcomitrella patens*	[14]
*CsLEA11*	Cold, Heat	Unknown	*Cucumis sativus*	[16]
*DHN-5*	ABA, Salt	Unknown	*Triticum durum*	[84]
*TaDHN1*	ABA, Salt	ABRE, HSE	*Triticum aestivum*	[91]
*TaDHN3*	ABA, Salt	ABRE, HSE	*Triticum aestivum*	[91]
*AmDHN1*	Dehydration, Salt	DRE, MYB binding element	*Avicennia marina*	[87]
*BjDHN2*	heavy metal	Unknown	*Brassica juncea*	[89]
*BjDHN3*	heavy metal	Unknown	*Brassica juncea*	[89]
*PvSR3*	heavy metal	Unknown	*Phaseolus vulgaris*	[88]
*COR15*	Cold	Unknown	*Brassica oleracea*	[95]
